# Efficacy of Repositioning Therapy in Patients With Benign Paroxysmal Positional Vertigo and Preexisting Central Neurologic Disorders

**DOI:** 10.3389/fneur.2018.00486

**Published:** 2018-06-29

**Authors:** Chih-Chung Chen, Hsiao-Shan Cho, Hsun-Hua Lee, Chaur-Jong Hu

**Affiliations:** ^1^Department of Neurology, School of Medicine, College of Medicine, Taipei Medical University, Taipei, Taiwan; ^2^Department of Neurology, Taipei Medical University–Shuang Ho Hospital, New Taipei City, Taiwan; ^3^Dizziness and Balance Disorder Center, Taipei Medical University–Shuang Ho Hospital, New Taipei City, Taiwan; ^4^Graduate Institute of Clinical Medicine, Taipei Medical University, Taipei, Taiwan

**Keywords:** benign paroxysmal positional vertigo, repositioning, Epley maneuver, barbecue roll maneuver, Gufoni maneuver, residual dizziness

## Abstract

With the exception of migraines, benign paroxysmal positional vertigo (BPPV) in patients with preexisting central neurologic disorders (CND) is rarely discussed in the literature. Demographic features of this patient group and the efficacy of repositioning therapy are still unknown. We hypothesized that a CND may alter the function of the central vestibular pathway, thus changing the pattern of BPPV and outcomes of repositioning. In this study, we enrolled 93 consecutive idiopathic BPPV patients and categorized them into two groups according to the presence or absence of a CND. In our series, 31.2% of BPPV cases had a CND. The most common associated CNDs were cerebrovascular disease and migraines. The two groups showed similar age distributions, canal involvement, success rates of repositioning, and cycles of treatment used to achieve complete resolution. The major differences were the proportion of females (89.7%) and a right-side predominance (75.9%) in the CND group. There was a trend of more residual dizziness (RD) after successful repositioning in the CND group, but the difference was not significant. The reason for the female and right-side predominance in the CND group is unclear. We concluded that the efficacy of repositioning therapy was excellent (with a success rate of 80.6% with one cycle and 93.5% within two cycles of treatment) for BPPV with or without a preexisting CND. Clinicians are encouraged to diagnose and treat BPPV in patients with a preexisting CND as early as possible to improve patients' quality of life, avoid complications, and reduce medical costs.

## Introduction

Benign paroxysmal positional vertigo (BPPV) is probably the most common cause of episodic vertigo (1). It is caused by dislodged otoconia moving into one or more semicircular canals. Two major types of BPPV have been identified—canalolithiasis and cupulolithiasis. In the case of canalolithiasis, a certain head position causes free particles to move in the canal due to the effect of gravity, and the endolymphatic flow is disturbed. In the case of cupulolithiasis, particles adhere to the cupula and render it a gravity-sensitive organ. Among the three canals, the posterior canal (PC) is most often involved, followed by the horizontal (HC) and anterior canals (AC). The characteristic presentation of BPPV is a short duration of intense spinning vertigo as soon as a patient lies down, turns the head to the side in bed, or rises up from a supine position. Sometimes, hyperextension or flexion of the neck in an upright posture can induce similar symptoms. Secondary BPPV implies that trigger factors are known, for example, recent head trauma or an inner ear disease. Although no trigger events can be identified in the case of idiopathic BPPV, risk factors such as old age, vitamin D deficiency, and osteoporosis have been proposed (2–4).

Positioning maneuvers in the standard clinical vestibular function test help diagnose BPPV without too much difficulty. The Dix-Hallpike maneuver uncovers typical vertical and torsional nystagmus in PC- and AC-BPPV; the supine head roll maneuver uncovers typical geotropic or apogeotropic horizontal nystagmus in HC-BPPV (5). Particle repositioning therapy is highly effective in treating BPPV. The Epley and Semont maneuvers are commonly used to treat PC-BPPV, and the Gufoni and Barbecue roll maneuvers are commonly used to treat HC-BPPV. The optimal treatment for AC-BPPV remains controversial (6).

Concerning cycles used in each repositioning procedure, various protocols have been proposed. Conventional treatment involves repetitive cycles until the positional nystagmus disappears (7). Owing to the high treatment efficacy, some protocols adopt single-cycle treatment in each repositioning procedure (8, 9). Single treatment may suffice to treat most cases; nevertheless, some patients may require multiple treatments. After successful repositioning, some patients still report residual symptoms (10). Patients may describe a discomfort sensation as non-spinning dizziness or unsteadiness. Differentiating residual dizziness (RD) from unsuccessful repositioning is possible by recording positional nystagmus through Frenzel goggles or video nystagmography (VNG). The pathogenesis of RD is still unclear. The following hypotheses have been proposed: (1) persistence of debris; (2) utricular dysfunction; (3) incomplete central adaptation; and (4) sympathoneural deregulation (11).

BPPV is so common in vertiginous patients that it is not surprising it may attack one with a preexisting central neurologic disorder (CND). Common CNDs, such as cerebrovascular disorders, migraines, epilepsy, Alzheimer's disease, multiple sclerosis (MS), and Parkinson's disease (PD), may present with symptoms of dizziness or vertigo at any time in their disease courses. On evaluating a dizzy patient with a CND, the origin of the dizziness is often explained by the CND itself or as an adverse effect of medication. Therefore, diagnosing BPPV in patients with a preexisting CND is challenging, particularly for general neurologists. With the exception of migraines, papers discussing diagnosis and treatment outcomes of patients with both BPPV and CNDs are sparse. One study noted higher frequency of BPPV in giant cell arteritis than in normal controls and attributed the association to ischemic mechanism (12). Whether the success rate of repositioning treatment in BPPV with a CND is similar to that without a CND is unknown. In addition, whether the incidence of RD after treatment will be higher in patients with CNDs as a result of an altered central vestibular pathway and central adaptation needs to be investigated.

## Materials and methods

### Participants

The protocol of this study was approved by the Taipei Medical University—Joint Institutional Review Board (no. N201802092). Consecutive subjects visiting specialized neurologic clinics in Taipei Medical University–Shuang Ho Hospital (New Taipei City, Taiwan) from January 2015 to June 2017 with the main complaint of dizziness, vertigo, or imbalance were eligible candidates. After thorough history taking and a standardized clinical vestibular evaluation, tentative diagnoses of any of the following vertigo syndromes, including BPPV, vestibular migraine, vestibular neuritis, persistent postural-perceptual dizziness, Menière's disease, vestibular paroxysmia, bilateral vestibulopathy, cervicogenic dizziness, orthostatic intolerance, non-vestibular dizziness, and central vertigo disorders, were made. Appropriate laboratory audiovestibular tests or imaging studies were arranged to facilitate a final diagnosis, including pure-tone audiometry, vestibular evoked myogenic potentials, a full VNG study, caloric test, brainstem auditory evoked potential, posturography (Biodex balance system), tilting table test, sonographic study of extra- and intracranial arteries, cervical spine/brain computed tomography, or magnetic resonance imaging. A predesignated registry system was used to collect relevant clinical information of eligible candidates. For candidates with BPPV, the sex, age, preexisting CND, comorbid vestibular diagnoses, trigger events, involved side and canals, symptom duration, types and cycles of the repositioning procedure, canal conversion, RD after successful repositioning, and recurrence were recorded. Candidates with definite idiopathic BPPV who completed the clinical follow-up were enrolled in this study. Candidates with secondary BPPV, with multiple canal involvement, or who did not receive the repositioning procedure or complete the clinical follow-up were excluded. The institutional review board waived requirement for written informed consent because of the retrospective nature of the study.

### Standardized clinical vestibular evaluation

The standardized clinical vestibular evaluation in our protocol comprised the following assessments: eye alignment by cover test, smooth pursuit, saccades, spontaneous nystagmus with and without visual fixation by Frenzel goggles, gaze-evoked nystagmus, clinical head thrust in six planes, head-shaking nystagmus, positional nystagmus with Frenzel goggles, limb dysmetria, stance on firm or foamy surface with eyes open and closed, one-leg stance, tandem walking with eyes open and closed, and Unterburger's (Fukuda) test.

### Diagnosis of definite BPPV

BPPV was diagnosed by uncovering characteristic nystagmus during the positional test in a standardized clinical vestibular evaluation. Basically, no additional laboratory audiovestibular test or imaging study was needed to confirm the diagnosis. Some subjects received video recording of the positional nystagmus for confirmation of the diagnosis and follow-up comparisons. All of our study candidates fulfilled the diagnostic criteria of definite PC-, HC-, or AC-BPPV in the consensus document of the Committee for the Classification of Vestibular Disorders of the Bárány Society in 2015 (5).

### CNDs

Based on the report of public health challenges in neurological disorders by the World Health Organization in 2006, we selected CNDs of high disability-adjusted life years as our study target (13). The ranking of the global burden of disease projected for 2015 from high to low is cerebrovascular disease, dementia, migraines, epilepsy, meningitis, PD, and MS. With the exception of meningitis, all other diseases are chronic or recurrent in nature and are likely to present with dizziness, vertigo, or imbalance. At the time of the BPPV diagnosis, medical records were reviewed to document preexisting CNDs. Candidates were categorized into either the CND group or non-CND group.

### Repositioning procedure

We performed the repositioning procedure on the same day, usually within 1 h, of the BPPV diagnosis. All patients were given notes describing the BPPV pathogenesis, risk factors, treatment, and prognosis. Details of the repositioning procedure were explained with the aid of pictorial materials in the 2015 clinical practice guidelines from the American Academy of Otolaryngology—Head and Neck Surgery Foundation (AAO-HNSF) (14). Premedication for prevention of procedure-related nausea or vomiting was not used. We did not apply mastoid oscillation except in cases of apogeotropic HC-BPPV. For each patient, we used only one cycle of repositioning on the day of the visit. During the course of repositioning, clinicians observed the nystagmus through Frenzel goggles to decide the timing of the subsequent step. To treat PC-BPPV, we used the Epley maneuver. To treat geotropic HC-BPPV we used either the Gufoni or Barbecue roll maneuver. Details of the Epley, Gufoni, and Barbecue maneuvers are described in the AAO-HNSF guidelines (14). To treat apogeotropic HC-BPPV, we used the Gufoni maneuver for the apogeotropic variant described by Appiani et al. and added ipsilesional mastoid oscillation at the first step of the side-lying position to facilitate particle movement into the posterior limb of the HC (15). To treat AC-BPPV, we used either the reverse Epley or Yacovino maneuver (16, 17).

### Patient follow-up protocol

All patients were asked to maintain their normal daily activities without wearing a neck collar, but to avoid vigorous head shaking. On the first night after treatment, we suggested that every patient sleep with two pillows, although such a recommendation is not in current guidelines. We do not routinely prescribe vestibular suppressants after repositioning. All patients made an appointment to return to the clinic within 3 days if possible. On the follow-up visit, clinicians assessed the repositioning outcome by a subjective report and objective positional tests. Three levels of the subjective report were recorded: full, partial, and no response. Positional tests for all six canals with Frenzel goggles ensued regardless of the level of the subjective report. The objective outcome was categorized as either complete resolution, partial/no resolution, or canal conversion. Those who were categorized as partial/no resolution or canal conversion were treated with another cycle of an appropriate procedure, and the next visit was arranged within 3 days if possible. Successful repositioning was defined as complete resolution of any positional nystagmus. RD was defined as a subjective report of partial or no response despite successful repositioning. Treatment failure was defined as partial/no resolution after four or more cycles of repositioning.

### Analysis

The sex, age, symptom duration, side, involved canal, success rate, number of treatment cycles, and RD were analyzed for the entire cohort and the two groups individually. IBM SPSS Statistics 19 (Windows) was used for statistical computation. Student's *t*-test was used to compare the difference in the age distribution between the two groups. A Chi-squared test was used to compare differences in sex, side, involved canal, success rate, number of treatment cycles, and RD between the two groups. A significant difference was defined as *p* < 0.05. Inspired by Faralli et al. (18), the correlation of RD and symptom duration in the entire cohort was analyzed as well. Short, medium, and long symptom durations were respectively defined as intervals between onset and treatment of ≤4, 5–8, and ≥9 days.

## Results

**Figure 1:**
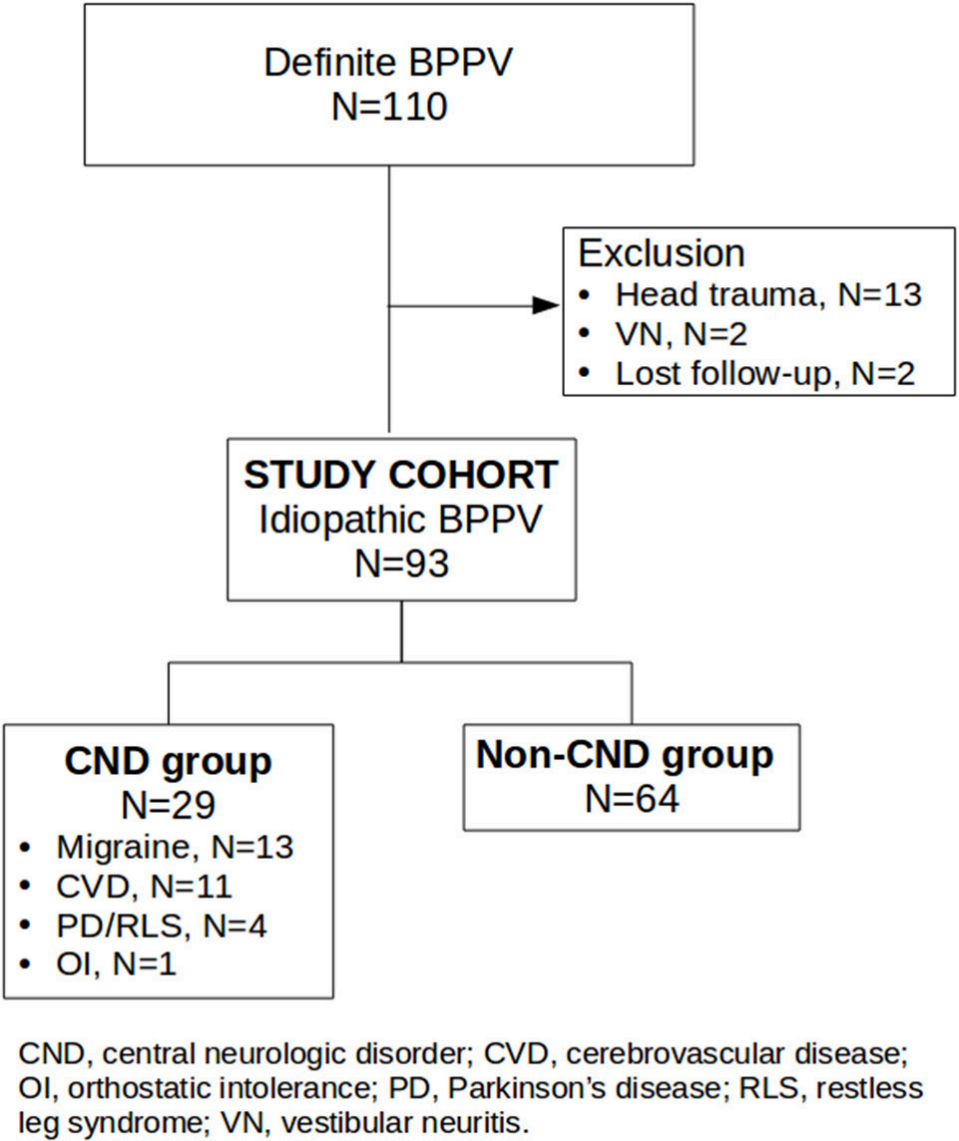
Flow chart of candidate recruitment and categorization. The study cohort comprised 93 idiopathic BPPV cases. The CND group comprised 29 cases, 13 with migraines, 11 with cerebrovascular disease, three with PD, one with restless leg syndrome, and one with idiopathic orthostatic intolerance. The non-CND group comprised 64 cases.

In total, 110 definite BPPV subjects were identified. Seventeen subjects were excluded from this study: thirteen secondary to head trauma, two secondary to vestibular neuritis, and two lost to follow-up. The remaining 93 subjects were enrolled and categorized into two groups (Figure 1). Analysis of the entire cohort (*N* = 93) showed a mean age of 63.2 [standard deviation (SD) 12.3] years, 63 females (67.7%), 56 on the right side (60.2%), 67 with PC (72.1%), 23 with HC (24.7%), three with AC (3.2%), four with canal conversion, two with treatment failure, a 97.8% overall success rate, and 32 with RD (35.2%). Cumulative success rates were 80.6% with one cycle and 93.5% within two cycles of treatment. Of 91 successfully treated cases, RD was present in 48.7% of patients with short, in 23.5% with medium, and in 25.7% with long symptom durations. Patients with short symptom duration were more likely to report RD [*p* < 0.02, odds ratio (OR) = 2.85]. The CND group comprised 29 cases (31.2%), 13 with migraines, 11 with cerebrovascular disease, three with PD, one with restless leg syndrome, and one with idiopathic orthostatic intolerance. The non-CND group comprised 64 cases (68.8%). Parameters of the two groups are summarized in Table 1 (Supplementary Material). Age distributions and symptom duration were similar between the two groups. Females outnumbered male patients in both groups, but the prevalence was significantly higher in the CND group (*p* < 0.01). A right-side predominance was noted in both groups, and the phenomenon was more significant in the CND group (*p* = 0.04). Canal involvement, the overall success rate, cumulative success rate within two treatment cycles, and recurrence did not differ between the two groups. Although the incidence of RD was slightly higher in the CND group, the difference was not significant. Interestingly, the success rate with one treatment cycle was higher in the CND group (*p* = 0.04). Further analysis of RD in patients with a short (≤4 days) symptom duration did not reveal a correlation with the presence or absence of a CND.

**Table 1:** Parameters of the two groups.

	**CND group**	**Non-CND group**	
	***N* = 29**	***N* = 64**	
Age (mean ± SD), years	64.6 ± 10.5	62.5 ± 13.1	*p* > 0.05
Females	26 (89.7%)	37 (57.8%)	***p*** < **0.01**
**SYMPTOM DURATION**			
≤ 4 days	10 (34.5%)	30 (46.9%)	*p* > 0.05
5~8 days	7 (24.1%)	10 (15.6%)	
≥ 9 days	12 (41.4%)	24 (37.5%)	
Right side	22 (75.9%)	34 (53.1%)	***p*** = **0.04**
**CANAL**			
Posterior canal	25	42	*p* > 0.05
Horizontal canal	4	19	
Anterior canal	0	3	
Successful treatment	28 (96.6%)	63 (98.4%)	*p* > 0.05
**CUMULATIVE SUCCESS RATE**			
With one cycle	93.1%	75.0%	***p*** = **0.04**
Within two cycles	96.6%	92.2%	*p* > 0.05
Residual dizziness	12 (42.9%)	20 (31.7%)	*p* > 0.05
Recurrence	0 (0%)	2 (3.1%)	*p* > 0.05

*CND, central neurologic disorder; SD, standard deviation. Bold values indicate significant difference*.

## Discussion

Diagnosing BPPV in patients with a preexisting CND is challenging, particularly for general neurologists. As described in the previous section, dizziness in these patients is often explained by the CND itself or an adverse effect of medication. Given that BPPV is a mechanical condition of the inner ear, one would expect similar diagnostic and treatment procedures even in the presence of a CND. However, the efficacy of repositioning therapy in this patient group was never discussed before.

With the exception of migraines, BPPV with a CND has not frequently been mentioned in the literature. In an early retrospective study by Ishiyama et al. 28.7% of idiopathic BPPV patients could also fit the IHS diagnostic criteria of migraines. They found that patients with idiopathic BPPV were 3-times more likely to have migraines than patients with secondary BPPV, and they proposed a shared pathogenesis of a vasospasm-induced metabolic abnormality (19). von Brevern et al. demonstrated a strong association of BPPV with migraines in their epidemiologic study (2). Later studies revealed that the efficacy of repositioning in BPPV with migraines was not inferior to that without migraines (20, 21). The link between BPPV and stroke is obscure. von Brevern et al. noted stroke as an independent risk factor of BPPV (2). A nationwide population study in Taiwan showed an increased risk of stroke in BPPV patients (22). Studies of BPPV treatment in PD, MS, and other CNDs are also sparse. A systemic analytical study by Wensen et al. examined 16 PD patients with BPPV (23). They found that these patients tended to have higher Dizziness Handicap Inventory scores, but the recovery 3 months after repositioning was good. In a study of 1153 MS patients, Frohman et al. concluded that the most common cause of isolated vertigo in MS patients was BPPV, followed by relapsing demyelinating plaques. The outcome of repositioning for BPPV in MS patients was good as well (24).

Two of the most common CNDs in our series were migraines and cerebrovascular disease. The case numbers of either were not large enough to have separate analyses, and that is the reason we grouped all CNDs together for our analysis. However, further inspection into both subgroups showed similar treatment outcomes regarding success rate and RD. Nearly 90% of CND group patients in this study were female. Sex distributions of the two groups were not comparable. In our series, all migraineurs and eight out of 11 stroke patients were female. Migraines were more prevalent in female than in male populations in the epidemiological studies. However, we did not have good explanations for female predominance in stroke patients with BPPV. We would like to see a study with a larger patient series to support our observation. Although side involvements did not affect the treatment outcomes, a significant right side predominance (75.9%) in CND group drew our attention. In most case series, right side involvements were more common than the left side. The widely accepted hypothesis is the preferential right-side lying position during sleep in susceptible subjects (25). Because we did not analyze the preference of lying side during sleep in this study, we could not evaluate the influence on side involvements in this group.

A high success rate of repositioning can be achieved in patients both with or without a CND, and numbers of cycles of the procedure needed for curing did not greatly differ. The findings are in accordance with the current theory of the BPPV pathogenesis. This also provides strong evidence to encourage clinicians to diagnose BPPV as early as possible in dizzy patients with a preexisting CND. Correct and prompt treatment in this patient group would surely improve the life quality, prevent complications, and reduce medical costs.

Over 80% of cases were free from positional nystagmus after a single treatment which is also in line with 68–90% success rates with the global single-cycle treatment experience (26). Interestingly, we noted a significantly higher success rate with single-cycle treatment in the CND group. Rather than implying that CNDs enhance the response of repositioning treatment, we doubted that a higher incidence of PC involvement in our CND cohort may have been the cause.

RD has a negative impact on the quality of life and the psychological status of BPPV patients. Risk factors, including aging, a long symptom duration, and anxiety were previously proposed, but study results are conflicting (10, 18, 27–31). Optimal management of RD remains unclear. Effects of etizolam, betahistine, dimenhydrinate, and repeated repositioning have been reported (32–35). Faralli et al. reported an increased chance of RD in migraineurs and attributed it to migraine-associated stress and anxiety (20). To test our hypothesis that CNDs alter the function of the central vestibular pathway, thus impairing central adaptation and increasing the chance of RD, we compared the incidence of RD between the two groups. Although we observed a higher percentage of RD in the CND group, the difference was insignificant. Therefore, the hypothesis that the CND group is more likely to have RD was not fully supported by our study. A larger case-control study in the future is needed to verify this finding. The findings of more frequent RD in patients with short (≤4 days) symptom durations in our study were different from previous studies (11, 18, 27–29). We assumed that symptom durations may not be the sole factor in the development of RD. One possible explanation was that patients who sought medical help at short symptom durations tended to be more anxious than those at long symptom durations.

The peculiarly high (31.2%) incidence of idiopathic BPPV and preexisting CNDs in our series can be explained in two ways. First, the nature of our registry system shaped the candidate pattern. The candidate source in this study was based on “specialized neurologic,” rather than neuro-otologic or otolaryngologic clinics, hence this caused the population to differ. Second, secondary BPPV was not included in this study. Taking all 110 BPPV cases into consideration, the incidence of BPPV with a preexisting CND was a little bit lower (30%).

We have to address some limitations of this study. First, lesions in CNDs and in the vicinity of the central vestibular pathway were not closely associated. In our series, a discrete brain lesion could not always be localized in CNDs, except for cerebrovascular disease. Second, we did not test the anxiety score of our patients, and so whether RD is related to anxiety could not be established. Third, sex distributions of the two groups were not comparable. Finally, the case number of the CND group was small. In addition, we did not include epilepsy or MS cases in our series.

## Conclusions

Evaluation of dizziness in patients with a preexisting CND is complex. A correct diagnosis and treatment of BPPV in these patients warrant rapid and predictable symptom resolution, and in the meantime, avoid unnecessary imaging tests and medications. In our series, about 30% of idiopathic BPPV patients had a preexisting CND, and their neurological conditions did not hinder successful repositioning in most instances. The efficacy of repositioning therapy was as good as for those without a CND. RD after successful treatment was slightly prevalent in this patient group, but the difference was not significant. Concerning symptom duration as a contributory factor to RD, we found that patients with a short symptom duration were more likely to have reported RD after successful repositioning treatment.

## Ethics statement

The protocol was approved by the Taipei Medical University—Joint Institutional Review Board and was performed in accordance with the ethical standards laid down in the 1964 Declaration of Helsinki.

## Author contributions

C-CC analyzed the data and drafted the manuscript. H-SC collected candidate information. H-HL and C-JH edited the manuscript.

### Conflict of interest statement

The authors declare that the research was conducted in the absence of any commercial or financial relationships that could be construed as a potential conflict of interest.

## Abbreviations

AC: anterior canal
BPPV: benign paroxysmal positional vertigo
CND: central neurologic disorder
HC: horizontal canal
MS: multiple sclerosis
PC: posterior canal
PD: Parkinson's disease
RD: residual dizziness
VNG: video nystagmography.

## References

[1] Nuti D Masini M Mandalà M Chapter 18: Benign paroxysmal positional vertigo and its variants. In: Furman JM Lempert T editors. Handbook of Clinical Neurology, Vol. 137 Amsterdam: Elsevier (2016). pp 241–56. 10.1016/B978-0-444-63437-5.00018-2 27638076

[2] von Brevern M Radtke A Lezius F Feldmann M Ziese T Lempert T Epidemiology of benign paroxysmal positional vertigo: a population based study. J Neurol Neurosurg Psychiatry (2007) 78:710–5. 10.1136/jnnp.2006.100420 17135456 PMC2117684

[3] Jeong SH Kim JS Shin JW Kim S Lee H Lee AY. Decreased serum vitamin D in idiopathic benign paroxysmal positional vertigo. J Neurol. (2013) 260 832–8. 10.1007/s00415-012-6712-2 23096068

[4] Yu S Liu F Cheng Z Wang Q. Association between osteoporosis and benign paroxysmal positional vertigo: a systematic review. BMC Neurol. (2014) 14:110. 10.1186/1471-2377-14-110 24886504 PMC4039044

[5] von Brevern M Bertholon P Brandt T Fife T Imai T Nuti D Benign paroxysmal positional vertigo: diagnostic criteria. J Vestibul Res. (2015) 25(3–4):105–17. 10.3233/VES-150553 26756126

[6] Anagnostou E Kouzi I Spengos K. Diagnosis and treatment of anterior-canal benign paroxysmal positional vertigo: a systematic review. J Clin Neurol. (2015) 11 262–7. 10.3988/jcn.2015.11.3.262 26022461 PMC4507381

[7] Epley JM. The canalith repositioning procedure: for treatment of benign paroxysmal positional vertigo. Otolaryngol Head Neck Surg. (1992) 107 399–404. 10.1177/019459989210700310 1408225

[8] Herdman SJ Tusa RJ Zee DS Proctor LR Mattox DE. Single treatment approaches to benign paroxysmal positional vertigo. Arch Otolaryngol Head Neck Surg. (1993) 119 450–4. 10.1001/archotol.1993.01880160098015 8457308

[9] Macias JD Lambert KM Massingale S Ellensohn A Ann Fritz J. Variables affecting treatment in benign paroxysmal positional vertigo. Laryngoscope (2000) 110 1921–4. 10.1097/00005537-200011000-00029 11081611

[10] Lee NH Kwon HJ Ban JH. Analysis of residual symptoms after treatment in benign paroxysmal positional vertigo using questionnaire. Otolaryngol Neck Surg. (2009) 141 232–6. 10.1016/j.otohns.2009.04.006 19643257

[11] Giommetti G Lapenna R Panichi R Mobaraki PD Longari F Ricci G. Residual dizziness after successful repositioning maneuver for idiopathic benign paroxysmal positional vertigo: a review. Audiol Res. (2017) 7:178. 10.4081/audiores.2017.178 28603599 PMC5452628

[12] Amor-Dorado JC Llorca J Costa-Ribas C Garcia-Porrua C Gonzalez-Gay MA. Giant cell arteritis: a new association with benign paroxysmal positional vertigo. Laryngoscope (2004) 114 1420–5. 10.1097/00005537-200408000-00020 15280720

[13] WHO (2006) Neurological Disorders: Public Health Challenges (ISBN: 978 92 4 156336 9) Available online at: http://www.who.int/mental_health/publications/neurological_disorders_ph_challenges/en/ (Accessed 1 Feburary 2018).

[14] Bhattacharyya N Gubbels SP Schwartz SR Edlow JA El-Kashlan H Fife T Corrigan MD. clinical practice guideline: benign paroxysmal positional vertigo (update). Otolaryngol Head Neck Surg. (2017) 156:S1–47. 10.1177/0194599816689667 28248609

[15] Ciniglio Appiani G Catania G Gagliardi M Cuiuli G Repositioning maneuver for the treatment of the apogeotropic variant of horizontal canal benign paroxysmal positional vertigo. Otol Neurotol. (2005) 26 257–60. 10.1097/00129492-200503000-00022 15793415

[16] Korres S Riga M Sandris V Danielides V Sismanis A. Canalithiasis of the anterior semicircular canal (ASC): treatment options based on the possible underlying pathogenetic mechanisms. Int J Audiol. (2010) 49 606–12. 10.3109/14992021003753490 20553103

[17] Yacovino DA Hain TC Gualtieri F. New therapeutic maneuver for anterior canal benign paroxysmal positional vertigo. J Neurol. (2009) 256 1851–5. 10.1007/s00415-009-5208-1 19536580

[18] Faralli M Lapenna R Giommetti G Pellegrino C Ricci G. Residual dizziness after the first BPPV episode: role of otolithic function and of a delayed diagnosis. Eur Arch Oto Rhino Laryngol. (2016) 273 3157–65. 10.1007/s00405-016-3947-z 26926693

[19] Ishiyama A Jacobson KM Baloh RW. Migraine and benign positional vertigo. Ann Otol Rhinol Laryngol. (2000) 109 377–80. 10.1177/000348940010900407 10778892

[20] Faralli M Cipriani L Del Zompo MR Panichi R Calzolaro L Ricci G. Benign paroxysmal positional vertigo and migraine: analysis of 186 cases. B-ENT (2014) 10 133–9. 25090812

[21] Yetiser S Gokmen MHA. Clinical aspects of benign paroxysmal positional vertigo associated with migraine. Int. Tinnitus J. (2015) 19 64–8. 10.5935/0946-5448.20150011 27186935

[22] Kao CL Cheng YY Leu HB Chen TJ Ma HI Chen JW Chan, R.-C. Increased risk of ischemic stroke in patients with benign paroxysmal positional vertigo: a 9-year follow-up nationwide population study in taiwan. Front Aging Neurosci. (2014) 6:108 10.3389/fnagi.2014.00108 24917815 PMC4040439

[23] van Wensen E van Leeuwen RB van der Zaag-Loonen HJ Masius-Olthof S Bloem BR Benign paroxysmal positional vertigo in Parkinson's disease. Parkin Relat Disord. (2013) 19 1110–2. 10.1016/j.parkreldis.2013.07.024 23948517

[24] Frohman EM Zhang H Dewey RB Hawker KS Racke MK Frohman TC. Vertigo in MS: utility of positional and particle repositioning maneuvers. Neurology (2000) 55 1566–9. 10.1212/WNL.55.10.1566 11094117

[25] Lopez-Escámez JA Gámiz MJ Fi-ana MG Perez AF Canet IS. Position in bed is associated with left or right location in benign paroxysmal positional vertigo of the posterior semicircular canal. Am J Otolaryngol. (2002) 23 263–6. 10.1053/ajot.2002.124199 12239689

[26] Reinink H Wegner I Stegeman I Grolman W. Rapid systematic review of repeated application of the epley maneuver for treating posterior BPPV. Otolaryngol Head Neck Surg. (2014) 151 399–406. 10.1177/0194599814536530 24876167

[27] Seok JI Lee HM Yoo JH Lee DK. Residual dizziness after successful repositioning treatment in patients with benign paroxysmal positional vertigo. J Clin Neurol. (2008) 4 107–10. 10.3988/jcn.2008.4.3.107 19513312 PMC2686873

[28] Faralli M Ricci G Ibba MC Crognoletti M Longari F Frenguelli A J Otolaryngol. (2009) 38 375–80. 10.2310/7070.2009.080073 19476771

[29] Teggi R Giordano L Bondi S Fabiano B Bussi M. Residual dizziness after successful repositioning maneuvers for idiopathic benign paroxysmal positional vertigo in the elderly. Eur Arch Oto Rhino Laryngol. (2011) 268 507–11. 10.1007/s00405-010-1422-9 21069369

[30] Martellucci S Pagliuca G de Vincentiis M Greco A De Virgilio A Nobili Benedetti FM Gallo A. Features of residual dizziness after canalith repositioning procedures for benign paroxysmal positional vertigo. Otolaryngol Neck Surg. (2016) 154 693–701. 10.1177/0194599815627624 26861236

[31] Seo T Shiraishi K Kobayashi T Mutsukazu K Fujita T Saito K. Residual dizziness after successful treatment of idiopathic benign paroxysmal positional vertigo originates from persistent utricular dysfunction. Acta Oto Laryngol. (2017) 137 1149–52. 10.1080/00016489.2017.1347824 28681630

[32] Jung HJ Koo JW Kim CS Kim JS Song JJ. Anxiolytics reduce residual dizziness after successful canalith repositioning maneuvers in benign paroxysmal positional vertigo. Acta Oto Laryngol. (2012) 132 277–84. 10.3109/00016489.2011.637179 22201336

[33] Guneri EA Kustutan O. The effects of betahistine in addition to epley maneuver in posterior canal benign paroxysmal positional vertigo. Otolaryngol Head Neck Surg. (2012) 146 104–8. 10.1177/0194599811419093 21852389

[34] Kim MB Lee HS Ban JH. Vestibular suppressants after canalith repositioning in benign paroxysmal positional vertigo. Laryngoscope (2014) 124 2400–3. 10.1002/lary.24741 24782447

[35] Tirelli G Nicastro L Gatto A Tofanelli M. Repeated canalith repositioning procedure in BPPV: effects on recurrence and dizziness prevention. Am J Otolaryngol. (2017) 38 38–43. 10.1016/j.amjoto.2016.09.009 27806891

